# Genes Related to Oxytocin and Arginine-Vasopressin Pathways: Associations with Autism Spectrum Disorders

**DOI:** 10.1007/s12264-017-0120-7

**Published:** 2017-03-10

**Authors:** Rong Zhang, Hong-Feng Zhang, Ji-Sheng Han, Song-Ping Han

**Affiliations:** 1 Neuroscience Research Institute, Peking University, Beijing, 100191 China; 2 Department of Neurobiology, School of Basic Medical Sciences, Peking University Health Science Center, Beijing, 100191 China; 3 Key Laboratory for Neuroscience, Ministry of Education/National Health and Family Planning Commission, Peking University, Beijing, 100191 China; 4 Fujian Provincial Key Laboratory of Neurodegenerative Disease and Aging Research, Institute of Neuroscience, College of Medicine, Xiamen University, Xiamen, 361005 China

**Keywords:** Oxytocin, Arginine-vasopressin, Single-nucleotide polymorphisms, Autism spectrum disorder

## Abstract

Autism spectrum disorder (ASD) is a highly heritable neurodevelopmental disorders characterized by impaired social interactions, communication deficits, and repetitive behavior. Although the mechanisms underlying its etiology and manifestations are poorly understood, several lines of evidence from rodent and human studies suggest involvement of the evolutionarily highly-conserved oxytocin (OXT) and arginine-vasopressin (AVP), as these neuropeptides modulate various aspects of mammalian social behavior. As far as we know, there is no comprehensive review of the roles of the OXT and AVP systems in the development of ASD from the genetic aspect. In this review, we summarize the current knowledge regarding associations between ASD and single-nucleotide variants of the human OXT-AVP pathway genes *OXT*, *AVP*, AVP receptor 1a (*AVPR1a*), OXT receptor (*OXTR*), the oxytocinase/vasopressinase (*LNPEP*), and ADP-ribosyl cyclase (*CD38*).

## Introduction

Autism spectrum disorder (ASD) is a pervasive neurodevelopmental disorders involving deficits in social interaction and social communication, as well as the presence of restricted interests and repetitive and stereotypic patterns of behavior. The estimated prevalence of ASD based on the 2014 National Health Interview Survey was 2.24%, more than three-fold increase since 2000 [1]. The rapid increase of ASD cases has stimulated research in recent decades. However, the etiology of ASD remains obscure, partly because of its etiological heterogeneity. Rather than a single causative factor, the combined effects and interplay between genetic heritability and environmental risk factors may be more important in the etiology of ASD. However, it is generally accepted that the etiology can, at least, be partly explained by genetic studies [2]. Specifically, studies in twins have shown a high concordance among homozygous twins (70%–90% [3]), which is much lower in discordant twins [4, 5]. The risk for a newborn child is >10-fold higher if a previous sibling has an ASD [6]. Family-based association testing (FBAT) and population-based case-control tests have increased knowledge about the genetic causes of ASD. Known variants conferring susceptibility include single-nucleotide variants, short insertions and deletions, and genomic copy-number variants [3]. Based on studies using quantitative molecular genetic techniques, the proportion of ASD explained by common genotyped single-nucleotide polymorphisms (SNPs) is estimated to be 17%–60% [7]. Therefore, the contribution of common variants to ASD is important and should not be neglected.

Oxytocin (OXT) and arginine vasopressin (AVP) are closely-related nonapeptides that only differ in two amino-acids and originate from separate genes derived from the duplication of a common ancestral gene [8]. In the vertebrate brain, both OXT and AVP are mainly synthesized in the paraventricular and supraoptic nuclei and processed along the axonal projections to the posterior lobe of the pituitary, where they are stored in secretory vesicles and released into the peripheral circulation. Besides, they are also released from dendrites and somata within the brain. In addition, these neurons project directly to other brain regions including the amygdala, striatum, hippocampus, bed nucleus of the stria terminalis, and the suprachiasmatic nucleus [9]. Recently, they have become increasingly attractive as potential therapeutic targets in the context of ASD research due to their regulatory roles in social preference, social behaviors, and recognition, as revealed by studies in both humans [9] and rodents (reviewed by Lukas and Neumann [10]). OXT and AVP function as “social factors” in the brain via binding to their corresponding receptors: the OXT receptor (OXTR) and AVP receptor 1A (AVPR1A). Evidence suggests that malfunction of these receptors is involved in the pathogenesis of ASD [11, 12]. CD38 is a nicotinamide adenine dinucleotide ectoenzyme that plays a role in hormone secretion and cell proliferation, differentiation, and migration [13]. Interestingly, this protein is highly expressed in the brain, plays an obligatory role in the central release of OXT [14] and is relevant to the development of ASD [15].

In this review, we focus on the associations between ASD and polymorphisms of genes encoding the elements of the OXT-AVP neuronal pathways OXT (*OXT/neurophysin*-*I*) and AVP (*AVP/neurophysin*-*II*), their receptors (*OXTR* and *AVPR1a*), *CD38*, and oxytocinase/vasopressinase (*LNPEP*), a peptidase responsible for the degradation of OXT and AVP into shorter peptides [16] (summarized in Table 1).

**Table 1:** Polymorphisms of genes encoding elements of the OXT and AVP pathways that are associated with ASD and autistic symptoms.

Genes	Year	Design	Sample size	Ethnicity	Significant polymorphism	Refs.
*OXT*	2009	Family	149 families	Israeli	rs6133010	[22]
	2014		1771 children	Swedish	rs2770378	[23]
	2016	Family	156 families	Not specified	rs6084258, rs6133010 and rs2740204	[25]
*OXTR*	2005	Family	195 families	Han Chinese	rs2254298, rs53576	[35]
	2007	Family	57 families	Caucasian	rs2254298	[37]
	2008	Family	133 families	Israeli	rs2268494, rs1042778	[38]
	2010	Family	215 families	Japanese	No	[39]
	2010	Case-control	280 cases, 440 controls	Japanese	rs237887, rs2264891, rs2254298, rs2268495	[39]
	2010	Family	199 families	Caucasian	No	[44]
	2010	Family	100 families	Caucasian	rs2270465	[45]
	2011	Family	1238 families	Caucasian	rs2268493, rs1042778, rs7632287	[43]
	2013	Case-control	132 cases, 248 controls	Japanese	rs35062132-G	[42]
	2014	Case-control	76 cases, 99 controls	Swiss	rs2254298, rs53576	[36]
	2014	Case-control	118 cases, 412 controls	Caucasian	rs2268493	[41]
	2015		105 cases	Japanese	28 variants	[46]
	2015 (a meta-analysis)	Family and case-control	2525 families, 454 cases, 595 control	Han Chinese, Israeli, Caucasian, Japanese	rs7632287, rs237887, rs2268491, and rs2254298	[11]
	2016	Family	175 families	German	rs237889-A	[40]
*AVPR1a*	2002	Family	115 families	Caucasian, African- and Asian-American	RS3	[72]
	2004	Family	65 families	Not specified	RS1 and RS3	[12]
	2006	Family	116 families	Not specified	Haplotype RS1-RS3-AVR	[73]
	2010	Family	148 families	Korean	RS1 and RS3	[74]
	2011	Family	177 families	Irish	RS1 (short alleles), rs11174815	[75]
	2015	Family	205 families	Finnish	RS1 (short alleles), Haplotype rs7307997-rs1042615, and RS3-rs1042615	[76]
*AVPR1b*	2016	Family	207 families	Caucasian, African- and Asian-American	rs35369693 and rs28632197	[78]
*CD38*	2010	Family	104 families	Caucasian	rs6449197, rs3796863	[66]
	2010	Family	170 families	Israeli	rs3796863, rs3796878, rs3796867, rs4516711, rs10805347, rs1803404, rs1130169	[15]
	2010	Family	188 families	Japanese	–	[66]
	2014		1771 children	Swedish	rs6449182	[23]

*OXT*, oxytocin; *OXTR*, oxytocin receptor; *AVPR1a*, AVP receptor 1a; *AVPR1b*, AVP receptor 1b; *CD38*, cyclic ADP ribose hydrolase; RS1 and RS3, promoter microsatellites of *AVPR1a*.

### *OXT* and *AVP*

The human *OXT*-*neurophysin I* (*NPI*) and *AVP*-*neurophysin II* (*NPII*) loci are closely linked at chromosome 20p13, separated by only 12 kb of intergenic sequence, and are oppositely transcribed [17]. This type of genomic arrangement could result from the duplication of a common ancestral gene followed by the inversion of one of them [18]. The *OXT*-*NPI* gene encoding the OXT prepropeptide consists of three exons: the first encodes several peptides including a translocator signal, the nonapeptide hormone, the tripeptide processing signal, and the first 9 residues of neurophysin; the second encodes the central part of neurophysin; and the third exon encodes the C-terminal region of neurophysin [19]. The OXT prepropeptide undergoes cleavage and other modifications as it is transported along the axon to the terminals. The mature products OXT and its carrier molecule neurophysin I, are provisionally stored in the axon terminals until neural inputs elicit their release. *AVP*-*NPII* has almost the same gene structure and post-translational processing as *OXT*-*NPI* [20].

A linkage study by Allen-Brady and colleagues provisionally identified a susceptibility locus for ASD near the *OXT*-*NPI* gene region that met the genome-wide significance criteria [21]. In addition, Ebstein *et al*. reported nominal associations between ASD and *OXT* rs6133010, as well as the haplotypes in 170 individuals with ASD [22]. At the behavioral level, investigators found an association between *OXT* rs2770378 and autism-like traits including language impairment and restricted behaviors in females with ASD [23]. In a study of ASD and hormonal genes, two SNPs in the *OXT*-*NPI* gene region were examined and a single SNP, rs2740204, was associated with stereotyped behavior but not overall diagnosis in the 177 probands with ASD [24]. A recent study has also shown that various SNPs (including rs6084258, rs6133010, and rs2740204) near the *OXT* and *AVP* genes are associated with a diagnosis of ASD, social behaviors, restricted and repetitive behaviors, and intelligence quotient (IQ), as well as plasma OXT level [25].

Interestingly, in healthy individuals, polymorphisms near or within the *OXT* gene are also associated with phenotypes of brain function in social interactions such as empathy [26], maternal behaviors (breast-feeding [27] and maternal vocalization [28]) and social anxiety [29].

### *LNPEP*

The OXT and AVP peptides have a half-life of ~20 min in cerebrospinal fluid [30] and 3 min in plasma [31]. When released centrally they are degraded within brain tissue by LNPEP, also referred to as placental leucine aminopeptidase, which preferentially degrades OXT and is thus regarded as an oxytocinase [16]. The enzyme also effectively degrades vasopressin and angiotensin III. LNPEP is detectable in various brain regions including the basal ganglia, cerebral cortex, and cerebellum [32]. In these regions, immunoreactive staining of LNPEP is specific for neurons, and not non-neuronal cells [32].

As far as we know, there is only one published study on *LNPEP* variants. The investigators found that the SNPs rs18059 and rs4869317 are associated with 28-day mortality in patients with septic shock. Moreover, the rs4869317 TT genotype is associated with increased plasma vasopressin clearance [33]. Although there has been no direct evidence for the involvement of LNPEP in altered human behavioral phenotypes, we speculate that this aminopeptidase may play a regulatory role in human social behaviors via influencing the central OXT and/or AVP levels and perhaps is a target for drug intervention in some disorders with social defects, such as ASD.

### *OXTR*

In the brain, OXT regulates a variety of social behaviors via binding to its sole receptor OXTR in various regions. The *OXTR* gene is present in a single copy in the human genome and has been mapped to the gene locus 3p25-3p26.2. The gene spans 17 kb, contains 3 introns and 4 exons [34], and encodes a 389-amino-acid polypeptide belonging to class I of the G protein-coupled receptor family [18].

*OXT* as a genetic risk factor for ASD is also supported by linkage analysis and disease association with common variants in *OXTR*. In a study involving Han Chinese individuals, Wu *et al*. [35] used the FBAT and found a significant genetic association between ASD and two *OXTR* SNPs, rs2254298 and rs53576. A number of haplotypes constructed with two, three, or four markers, particularly those involving rs53576, were significantly linked to ASD [35]. Nyffeler *et al*. [36] also found similar associations in a Caucasian population with high-functioning autism. Jacob *et al*. [37] replicated the study of Wu *et al*. in a Caucasian sample with a strictly-defined autistic disorder. Interestingly, the SNP rs2254298 but not rs53576 was found to be associated with ASD. Moreover, over-transmission of the G-allele to probands with ASD was reported, which was inconsistent with a previous study in a Han Chinese population. Lerer *et al*. [38] conducted a comprehensive study examining all the tagged SNPs across the *OXTR* gene region. As expected, significant associations were found for single SNPs and haplotype with ASD. Notably, these polymorphisms of *OXTR* showed significant associations with IQ and the Vineland Adaptive Behavior Scales for ASD. In a Japanese population, Liu *et al*. [39] analyzed 11 *OXTR* SNPs but did not detect any significant signal in the FBAT test. However, case-control analysis revealed significant associations between four SNPs and ASD. The most significantly associated SNP was rs2254298 with “A” as the risk allele [39]. This result was similar to those in a Han Chinese population, but in contrast to the observations in Caucasians. The ethnic difference in the linkage disequilibrium structure between Asian and Caucasian populations may contribute to the difference in the role of *OXTR* polymorphisms in ASD in the two populations. A recent meta-analysis of 16 *OXTR* SNPs including 3941 individuals with ASD from 11 independent samples [11] revealed associations between ASD and the *OXTR* SNPs rs7632287, rs237887, rs2268491, and rs2254298. *OXTR* was also associated with ASD in a gene-based test. These results are the most comprehensive examination of the association of common *OXTR* variants with ASD to date. Furthermore, Kranz *et al*. [40] tested two additional *OXTR* SNPs (rs237889 and rs237897) for association with ASD in German cohorts and found nominal over-transmission for the minor A allele of variant rs237889G>A. Di Napoli *et al*. [41] focused on Asperger Syndrome, a subgroup of ASD, and discovered a significant association with rs2268493 in *OXTR*. Ma *et al*. [42] reported that the G allele of variant rs35062132C>G was correlated with an increased likelihood of ASD. Further cell experiments showed that rs35062132C>G accelerates OXT-induced receptor internalization and recycling, indicating a functional variant.

However, *OXT* SNPs were not always associated with ASD in the association studies, especially when adjustment was made for multiple comparisons. Campbell *et al*. [43] examined 25 genetic markers spanning the *OXTR* locus in a relatively large American sample, and an association of the three markers rs7632287, rs2268493, and rs1042778 was found. However, all the significant associations disappeared after correction for multiple testing. Similarly, in a combined sample from Ireland, the UK, and Portugal, the findings of Wu *et al*. [35] and Jacob *et al*. [37] were not replicated, with no marker survived for association with ASD [44]. In addition, Wermter *et al*. [45] genotyped 22 SNPs in the *OXTR* genomic region in 100 families with high-functioning and atypical ASD, and found no association after correction for multiple comparisons.

Research focusing on epigenetic modifications and rare variations of the *OXTR* may provide additional evidence for a role of this gene in ASD. In 105 ASD individuals from Japan, investigators identified 28 novel variants including potential functional variants in the intron region and one rare mis-sense variant (R150S) [46]. Gregory *et al*. [47] examined copy number variations and epigenetic changes in the *OXTR* gene, and interestingly revealed that a genomic deletion containing the *OXTR* gene was present in an autistic proband. DNA methylation analysis indicated that the promoter region of *OXTR* is hypermethylated in independent datasets of individuals with autism as compared to control samples, in both peripheral blood mononuclear cells and temporal cortex. In healthy adults, *OXTR* methylation has been associated with activity in the dorsal anterior cingulate cortex and temporal parietal junction, regions strongly associated with social perception [48].

In healthy populations, SNPs across the human *OXTR* gene have been associated with pair-bonding behaviors [49], parenting [50, 51], face-recognition skills [52, 53], and emotional and cognitive empathy [54, 55]. Neuroimaging studies have shown that carriers of the *OXTR* rs53576 AA allele have a smaller volume and reduced functional connectivity of the hypothalamus [56, 57], and GG homozygotes have an increased local volume in the left hippocampus and amygdala [58], which indicates an association between *OXTR* genetic variation and structural and functional variability in brain regions relevant to social cognition. In addition, rs53576 GG homozygotes are more responsive to intranasal OXT administration. For example, OXT administration increases preference for infants’ faces [59] and social cooperation [60] among rs53576 GG homozygotes but not in A allele carriers. The most plausible mechanism by which *OXTR* SNPs influence the effects of OXT is through altering expression of the OXTR. In prairie voles, one non-coding polymorphism in the *Oxtr* (SNP2) explains the variance in OXTR expression in particular brain regions [61]. Specifically, T-allele genotypes of SNP2 have double the OXTR density in the nucleus accumbens than CC littermates.

### *CD38*

Further evidence for an important role of the OXT system in ASD comes from studies on CD38, a transmembrane protein involved in OXT release in the brain [62] and in the critical regulation of social behavior [14, 63]. *Cd38*-knockout mice show severe social deficits (i.e., amnesia of conspecifics) and have been discussed as a rodent model of ASD [64, 65]. In individuals with ASD, two SNPs of *CD38* (rs6449197 and rs3796863) have been associated with high-functioning autism in the US population [66]. These findings were partially confirmed in Israeli participants [15], but not in Japanese cases [66]. For the rs3796863 SNP, ASD patients carrying the CC genotype are characterized by more severe symptoms, such as restricted, repetitive, and stereotyped patterns of behavior, than those carrying the A allele [66].

In healthy populations, individuals homozygous for the CC allele on *CD38* rs3796863 show a lower level of peripheral OXT than CA/AA carriers [67, 68]. When exposed to social stimuli, healthy men with the CC allele show slower reaction-times and higher activation of the left fusiform gyrus [69], an area widely discussed in ASD research. At the behavioral level, parents with high-risk alleles have been shown to touch their infants less during a free-play session, and low-risk *CD38* alleles predict longer durations of parent-infant gaze synchrony [67].

Besides the SNPs, a mis-sense mutation (4693C>T) of *CD38* has been found in 0.6%–4.6% of a Japanese population and was associated with ASD in a case-control study [66]. Partial deletion of *CD38* has also been reported in a patient with autism and asthma [70]. Furthermore, autistic individuals also show low expression of CD38 in lymphoblastoid cells (LBCs) [15]. In LBCs, treatment with all-*trans* retinoic acid (a known inducer of CD38 [69]) reverses CD38 mRNA expression [71]. Such a demonstration may provide *in vitro* “proof of principle” that CD38 is a potential target in the clinical treatment of ASD.

### *AVPR1a*

In contrast to only one form of OXTR, there are three subtypes of AVPR, AVPR1a, AVPR1b, and AVPR2, which are all G-protein-coupled receptors. Of those, AVPR1a is predominantly expressed in the brain and is the most strongly implicated in neuropsychiatric phenotypes. Therefore, in this section, we mainly summarize associations between polymorphisms of *AVPR1a* and ASD.

Various studies have established possible associations between polymorphisms in the promoter region of the *AVPR1a* gene and autism phenotypes. The human *AVPR1a* promoter region contains two microsatellite repeats, RS1 and RS3, in the 5′ flanking region. Of these, RS3 is a complex repeat located 3625 bp upstream of the transcription start site, and RS1 is a (GATA)_*n*_ repeat located 553 bp upstream of the start site [9]. The first genetic study of *AVPR1a* and human behavior was conducted by Kim *et al*. [72], who showed a nominally significant transmission disequilibrium between an *AVPR1a* microsatellite (RS3) and ASD, but this association was not significant after Bonferroni correction. Later, Wassink *et al*. [12] also found significant disequilibrium with both RS1 and RS3 but in cases with less severe impairment of language. More recently, Yirmiya *et al*. [73] failed to find associations of specific *AVPR1a* alleles with ASD, but significant associations of haplotypes consisting of RS1, RS3, and an intronic microsatellite (AVR). In addition, significant associations have been reported between these three microsatellite haplotypes and social phenotypes of ASD. Another study genotyped 148 Korean trios (a family with parents and a child) and also found evidence for associations between *AVPR1a* microsatellites (RS1 and RS3) and ASD [74]. In a study of an Irish population, a weak association was found between short alleles of RS1 and the SNP rs11174815 and ASD [75]. Recently, a Finnish study analyzed the association of three microsatellites (RS1, RS3, and AVR) and 12 tagged SNPs in the promoter and coding regions of *AVPR1a*, and found that the best association was located in RS1 [76]. Promoter analysis predicted one potential binding site for MEF2C (myocyte enhancer factor 2C) at RS1, which may be involved in autistic behavior [77]. In addition, the *AVPR1b* SNPs rs35369693 and rs28632197 have been associated with ASD, and the significance remained after correction for multiple comparisons [78]. This was the first study reporting associations between *AVPR1b* SNPs and ASD.

These findings provide evidence for a contribution of genetic polymorphisms of *AVPR1a* to the risk for ASD, which is further supported by the social impairment found in mice lacking functional *Avpr1a* [79]. Interestingly, microsatellite repeats are also found upstream of *Avpr1a* in prairie voles, a commonly-used animal model for affiliative social behavior related to neuropeptide signaling [80]. In this type of animal, microsatellite length causes intraspecific variation in *Avpr1a* expression and, consequently, social behavioral traits [81].

In individuals who have developed normally, long *AVPR1a* RS3 repeats are associated with higher expression of hippocampal *AVPR1a* [82] than in those carrying short RS3 repeats. In addition, longer alleles of RS3 are associated with a higher level of economic altruism [82] and a greater level of prepulse inhibition [83], which is an indicator of social cognition. Moreover, polymorphisms of RS3 are also linked to adulthood social interaction [84], pair-bonding [85], trust behavior [86], and non-clinical autism spectrum phenotypes [87] in healthy individuals.

### *OXTR* Gene Polymorphisms and Efficacy of OXT Administration

Since OXT is closely associated with a series of social behaviors, the neuropeptide is regarded as a potential agent for ASD treatment [9, 88–93]. Accumulating evidence has suggested that exogenous OXT administration is beneficial for the remission of autistic symptoms by improving cooperation and a sense of trust [94], as well as enhancing social responsiveness [95, 96] and social reciprocity [97, 98]. However, several studies failed to replicate the beneficial clinical effects of OXT on ASD [99, 100]. We speculate that these inconsistent findings may be at least partly associated with genetic polymorphisms of *OXTR*. Because intranasally-administered OXT is considered to act through the OXTR [18] and the latter contains several dozen SNPs, the administered OXT would not be expected to have a pharmacological effect if there is a loss-of-function mutation in *OXTR*. Therefore, the efficacy of OXT administration might differ according to *OXTR* gene polymorphisms.

Animal studies have suggested that some *OXTR* SNPs contribute to individual differences in OXTR expression, but only in particular brain regions [61]. A single-dose study in healthy volunteers showed that *OXTR* gene polymorphisms alter the sensitivity to reward-relevant features and/or their aversive properties in infants [59] and also influence the improvement of neural responses associated with social cooperation [60]. With long-term OXT administration, ASD patients carrying a T-allele at rs6791619 of the *OXTR* show improved Clinical Global Impression-Improvement scores, providing direct evidence that *OXTR* SNPs are associated with the efficacy of OXT treatment [101]. Therefore, besides the regimen (e.g., dosage and number of administrations per day), participant characteristics including their genetic background are also important factors that need to be considered in clinical trials of OXT administration [102].

## Conclusions and Perspectives

In the current review, we summarize the key findings on associations between ASD and genetic polymorphisms of five genes that are key players in the architecture of the OXT-AVP neural pathways. We suggest that targeting elements of the OXT and AVP pathways is a potentially fruitful approach for drug discovery as well as a source of potential biomarkers for the early diagnosis of social disorders, especially ASD.

Animal studies suggest that epigenetic markers, including methylation and histone acetylation of the *OXTR*, are important in regulating the *OXTR* and *AVPR1a* genes [103, 104]. Notably, failure to examine the epigenetic modulation of OXT-pathway genes may be one reason for the lack of conclusive findings in a recent meta-analysis of *OXTR* rs53576 and rs2254298 [105]. Further investigations need to focus on not only the functional significance of *OXTR* SNPs but also potential epigenetic mechanisms, which will allow stronger and more comprehensive conclusions as to whether disruptions in oxytocinergic signaling contribute to a risk for ASD or are associated with variability in social deficiency in ASD.
